# Interspecific behavioural synchronization: dogs exhibit locomotor synchrony with humans

**DOI:** 10.1038/s41598-017-12577-z

**Published:** 2017-09-28

**Authors:** Charlotte Duranton, Thierry Bedossa, Florence Gaunet

**Affiliations:** 1 Laboratoire de Psychologie Cognitive, Aix-Marseille Université, CNRS, UMR7290, Fédération 3C, 3 Place Victor Hugo, CS 80249, Bât. 9, Case D, 13331, Marseille, CEDEX 03 France; 2 AVA Association, 40 Le Quesnoy, 76220 Cuy-Saint-Fiacre, France; 3 Ecole Nationale Vétérinaire d’Alfort, 7 Avenue du Général de Gaulle, 94704 Maisons-Alfort, France

## Abstract

Behavioural synchronization is widespread among living beings, including humans. Pairs of humans synchronize their behaviour in various situations, such as walking together. Affiliation between dyadic partners is known to promote behavioral synchronization. Surprisingly, however, interspecific synchronization has recived little scientific investigation. Dogs are sensitive to human cues, and share strong affiliative bonds with their owners. We thus investigated whether, when allowed to move freely in an enclosed unfamiliar space, dogs synchronize their behaviour with that of their owners’. We found that dogs visibly synchronized their location with their owner (staying in close proximity and moving to the same area), as well as their activity and temporal changes in activity (moving when their owner moved, standing still when their owner stood still, and gazing in the same direction as their owner). The present study demonstrates that owners act as attractors for their dogs in an indoor space, as mothers do for their children.

## Introduction

Behaving similarly to others is typical of many groups and dyads. Behavioural non-conscious synchronization is a widespread phenomenon, found in various taxa, such as insects, birds, and mammals; it has various adaptive values, such as increasing the efficiency of anti-predator strategies and increasing social cohesion (see ref.^1^ for a review). In non-human animals, synchronization is said to be non-conscious/implicit as there is no reliable method to demonstrate consciousness^2^; further, we do not consider here non-conscious synchronisation as a function of the optomotor reflex system^3^, as it is a complex behaviour that can be modulated by life experiences, such as attachement or learning - as described below.

Synchronization is indeed a broad term that encompasses different types of synchronies, such as temporal synchrony (switching actions at the same time, the actions can be identical or different, the important feature is the timing), location synchrony (being in the same place at the same time, the actions can be identical or different, the important feature is the localisation), and activity synchrony (exhibiting the same behaviour at the same time; for a review see refs^4,5^). All types of synchronies are present at the dyadic level, between two interacting individuals such as synchronization of swimming and breathing period in bottlenose dolphins (*Tursiops aduncus*)^6^, of bouts of vigilance in red-necked pademelons (*Thylogale thetis*)^7^, and of nest visiting in pairs of zebra finches (*Taeniopygia guttata*)^8^.

In humans, interpersonal interaction often results in the two partners coordinating/synchronizing their movements^9,10^. Synchronization is linked to affiliation between the partners: being synchronized strengthens social bonds between individuals, and conversely, the more affiliated two individuals are, the more they behave synchronously^11–13^. Synchronization is present in various situations, from rocking in rocking chairs^9^ to walking side by side^14,15^. The two latter studies investigated whether two people walking together would synchronize their behaviour even if they were not instructed to do so. The authors found that when walking together, the movements of each partner in a pair were not independent, but synchronized^15^. Social interaction with visual contact between the partners is thus sufficient to elicit behavioural synchronization, even in common activities such as walking together, and affiliation increases the degree of synchrony^9,16,17^.

One common situation in which humans walk with another individual is owners walking their dog. It has been suggested that synchronization of behaviour between humans and dogs can only emerge if there is attachment between the individuals, and that this synchrony relies on dogs ‘sensitivity to humans’ behavioural cues through previous learning experiences^18^. Current research would suggest that all conditions for synchronization between the two partners are present: dogs are well integrated into human societies, are highly sensitive to our behavioural cues (such as e.g. direction of attention) thanks to learning during life experiences, have typically developed strong affiliative bonds with humans (see ref.^4^ for a review), and are even proposed to resemble to their owners concerning stylistic attitudes/temperament^19^. However, the existence of temporal, location or activity synchrony between these two different species is poorly documented. Two studies have investigated dog-human behavioural synchronization while walking and yielded to the same conclusion. Guide dogs with their blind partner, as well as pet dogs with their blind-folded owner, presented non-conscious behavioural synchronization when walking, for instance in the start of movement or in the direction of walk^18^. During silent walks in the street, sighted owners and their dogs also presented synchrony in their direction and speed^20^. However, in both studies the majority of dogs were observed on leash (although in one study 6% were off-leash^20^). Therefore, it might be thought that, rather than non-conscious synchronization, most dogs observed had no choice but to synchronize their movements with those of their owners.

We decided to investigate the existence of behavioural synchronization between dogs and their owners when walking freely – i.e., without a leash. We tested dog-owner dyads moving in an enclosed unfamiliar room. We hypothesized that during a walk where both partners are physically free to move independently, the dogs would still synchronize their behaviour with that of their owners. More precisely, we hypothesized that the dogs would synchronize their location with their owner (i.e., move to the same part of the room and stay into close proximity with their owner). We also hypothesized that the dogs would synchronize their activity and switch of activity with their owner (i.e. walk if their owner walks, stop walking when their owner does, switching activities at the same time). Additionally, it has been found that when confronted with a stranger in an enclosed room, shepherd dogs remained more focused on their owners than molossoid dogs when the owner stayed still, but the difference disappeared when the owner was moving^21^. We thus also investigated potential effects of these breeds, as well as sex and age, on dogs’ behavioural synchronization.

## Results

All means and standard errors are presented in Table S2 and non-significant results in Table S3, in the Supplemental Material available.

### Location synchrony

#### Proximity to owner

The main aim of this study was to assess whether dogs modify their location in a room according to their owners’ location. The dogs spent an average of 23.84 ± 0.46 seconds within close range of their owners, for an average of 79.47% of total testing time. There was no effect of condition, breed, sex, or age on the amount of time that the dogs spent close to their owners (LMERs, *p* > 0.05 for all, see Table S3 in the Supplemental Material available).

#### Occupation of the room

There was no significant effect of breed, sex, or age on any of the following variables: time spent by the dogs in the centre of the room, on the right side of the room, or on the left side of the room (LMERs, *p* > 0.05 for all, see Table S3 in the Supplemental Material available).

**Figure 1:**
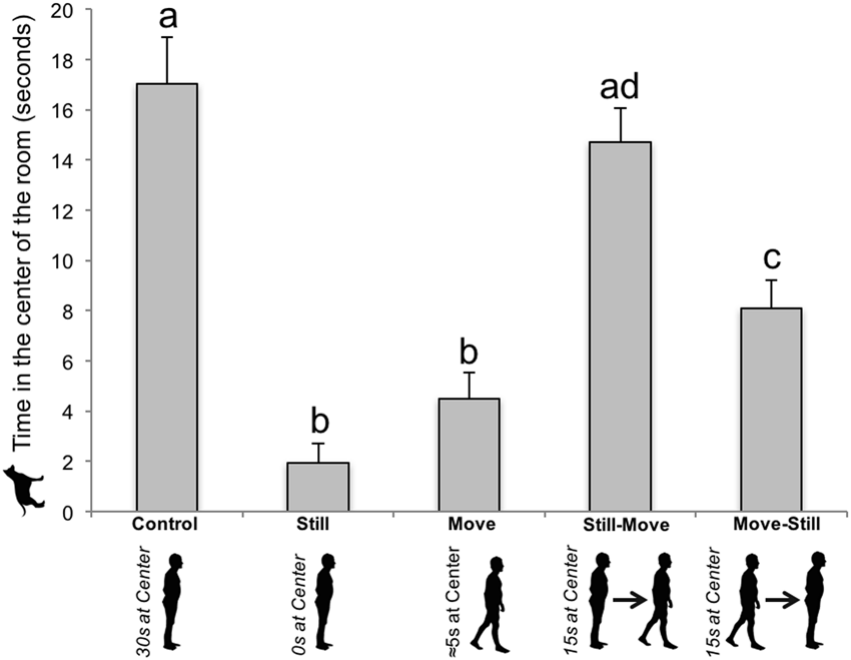
Time spent by the dogs in the centre of the testing room. Dogs (*N* = 48) spent significantly more time in the centre of the room in the control and still-move conditions compared to the other conditions. Different letters represent statistical differences. Data are presented as mean + SE.

Dogs spent significantly more time in the centre of the room in the control and still-move conditions compared to the other conditions (see Table 1 and Fig. 1)

**Table 1:** Significant results for the effect of testing conditions on dependant variables.

Dependant Variables	Results	Post-hoc comparisons	*χ* ^2^	Df	*P*	Cohen’s *d*	95% CI
Time in the centre	Overall effect	—	111.93	4	<0.001	—	—
	Post-hoc	Control/Still	58.20	1	<0.001	0.80	11.07–19.12
		Control/Move	35.19	1	<0.001	0.64	8.04–17.10
		Control/SM	1.41	1	0.23	0.14	−1.66–6.35
		Control/MS	17.45	1	<0.001	0.40	4.53–13.36
		Still/Move	3.71	1	0.054	0.12	−0.33–5.39
		Still/SM	77.19	1	<0.001	0.73	9.80–15.71
		Still/MS	23.52	1	<0.001	0.43	3.57–8.73
		Move/SM	45.95	1	<0.001	0.99	−13.29–−7.16
		Move/MS	6.17	1	0.01	0.30	0.64 – 6.60
		SM/MS	18.19	1	< 0.001	0.46	−9.75–−3.45
Time stationary	Overall effect	—	215.51	4	<0.001	—	—
	Post-hoc	Control/Still	0.00	1	0.98	0.00	−2.01–1.97
		Control/Move	111.80	1	<0.001	1.30	11.14–16.45
		Control/SM	31.74	1	<0.001	0.84	3.94–8.40
		Control/MS	51.13	1	<0.001	0.91	5.74–10.30
		Still/Move	141.49	1	<0.001	1.49	−16.18–−11.45
		Still/SM	26.70	1	<0.001	0.58	−8.63–−3.75
		Still/MS	50.63	1	<0.001	0.85	−10.33–−5.74
		Move/SM	54.40	1	<0.001	1.09	−9.72–−5.52
		Move/MS	24.87	1	<0.001	0.66	3.42–8.13
		SM/MS	3.23	1	0.07	0.22	3.93–0.24
Gaze to the front	Overall effect	—	76.22	4	<0.001	—	—
	Post-hoc	Control/Still	0.24	1	0.62	0.05	−1.91–3.13
		Control/Move	65.44	1	<0.001	0.90	5.61–9.38
		Control/SM	17.14	1	<0.001	0.46	2.14–6.29
		Control/MS	12.20	1	< 0.001	0.38	1.55–5.90
		Still/Move	50.29	1	<0.001	0.71	8.89–−4.88
		Still/SM	14.71	1	<0.001	0.47	−5.52–−1.69
		Still/MS	6.97	1	<0.01	0.26	−5.55–−0.68
		Move/SM	22.49	1	<0.001	0.53	−4.68–−1.87
		Move/MS	27.82	1	<0.001	0.48	2.31–5.22
		SM/MS	0.31	1	0.57	0.05	−1.30–2.28

Results of the LMERs are provided. All significant post-hoc comparisons were still significant after correction for multiple tests. Time in the centre = time spent by the dogs in the centre of the room. Time stationary = time spent by the dogs stationary. Gaze to the front = time spent by the dogs gazing to the front of the room. MS = Move-Still condition. SM = Still-Move condition. 95% CI and effect size, corresponding to Cohen’s *d*, are provided.

In this line, we found that the time the dogs spent in the centre of the room was significantly positively correlated with the time the owner spent in the centre of the room for all conditions pooled (Pearson’s correlation, *r* = 0.51, *p* < 0.001, 95% CI = [0.41–0.60]).

As the time the dogs spent on the right side of the room and time spent on the left of the room did not significantly differ (t-test, *p* = 0.39), we pooled these two variables together (see Table S1 in the Supplemental Material available). When dogs were not in the center of the room, they were on the sides; dogs’ time spent at the sides of the room is the complement of the dogs’ time spent in the centre of the room; the corresponding statistical results are provided in the Table S4 in the Supplemental Material available.

### Activity synchrony

Another aim of this study was to assess whether dogs modulated their activity according to that of their owners. Tests (LMERs) revealed no effect of breed, sex, or age on the dogs’ activity (time still, time moving, gaze direction; *p* > 0.05 for all, see Table S3 in the Supplemental Material available).

#### Locomotor activity

**Figure 2:**
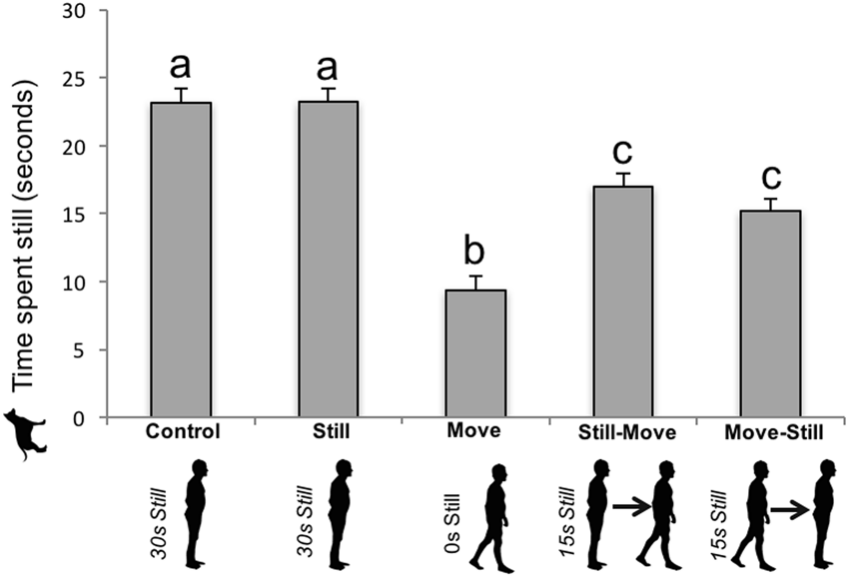
Dogs’ time spent stationary by experimental condition. Dogs (*N* = 48) spent significantly more time stationary in the control, still, and still-move conditions. Different letters represent statistical differences. Data are presented as mean + SE.

Dogs spent more time stationary in the control and still conditions than in the still-move, move-still, and move conditions (see Table 1 and Fig. 2). This is confirmed by a significant positive correlation between the time dogs spent stationary and the time the owners spent stationary (Pearson’s correlation for all conditions pooled: *r* = 0.60, *p* < 0.001, 95% CI = [0.51–0.68]).

Obviously, when dogs were not still, they were moving. When considering the total time of activity, dogs’ time spent moving is thus the complement of the dogs’ time staying still; the corresponding results are provided in the Table S4 in the Supplemental Material available.

#### Gazing activity

**Figure 3:**
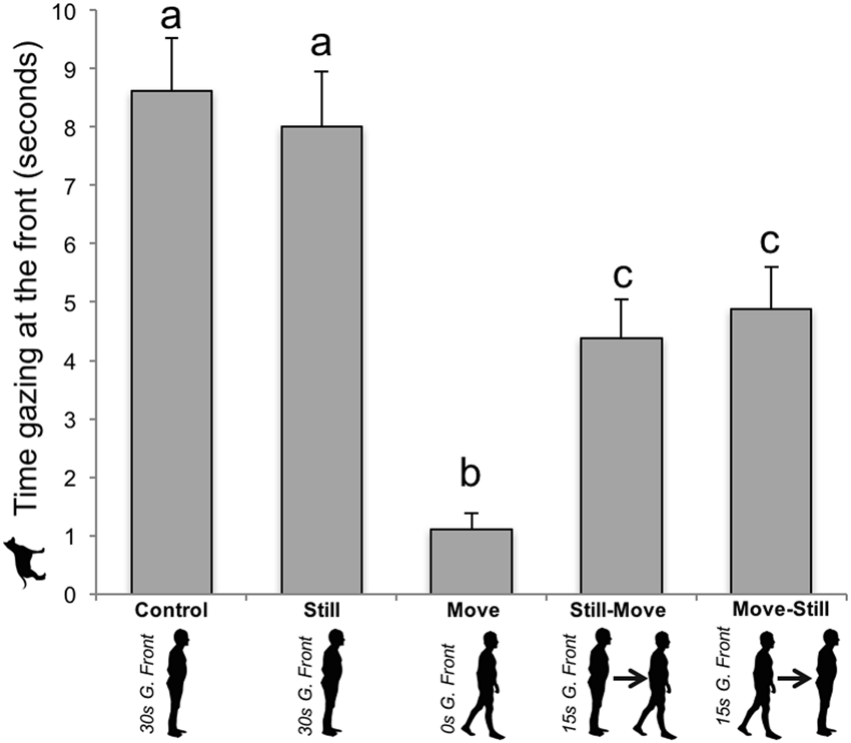
Dogs’ time spent gazing toward the front of the room, by experimental condition. Dogs (*N* = 48) spent significantly more time gazing toward the front of the room in the control and still conditions. Different letters represent statistical differences. Data are presented as mean + SE. G. = Gaze.

We found a significant effect of condition on the amount of time the dogs spent gazing toward the front of the room, with longer times in the control and still conditions than in the still-move, move-still, and move conditions (see Table 1 and Fig. 3).

The time the dogs spent gazing at the front of the room was significantly positively correlated with the time the owners spent gazing at the front of the room for all conditions pooled (Pearson’s correlation, *r* = 0.47, *p* < 0.001, 95% CI = [0.36–0.56]).

Time spent by the dogs gazing towards the sides of the room is presented in the Table S4 in the Supplemental Material available.

### Temporal synchrony

The final aim of this study was to assess whether dogs changed their activity when their owners’ changed theirs during the still-move and the move-still conditions. As the latency before dogs’ switched activity in both conditions did not significantly differ (t-test, *p* = 0.25), we pooled these two variables together (see the three definitions in Table S1 in the Supplemental Material available). In average, dogs switched their activity 3.40 ± 0.52 seconds after the owner switched their activity. Tests (LMs) revealed no effect of sex on the dogs’ latency before switching activity (*p* > 0.05, see Table S3 in the supplemental material). We found a breed effect, with shepherd dogs exhibiting a shorter latency (2.22 ± 0.44 seconds) before switching to the same activity as the owner compared to molossoid dogs (4.73 ± 0.93 seconds; LM, *χ* ^2^ = 70.95, Df = 1, *p* < 0.01, Cohen’s *d* = 0.76, 95% CI = [0.51–4.50]). Such a breed effect can easily be explained by physical issues: molossoids are generally heavier than shepherds, and weight is known to be linked to dogs’ velocity^22^. This would explain why molossoids needed more time to switch of activity. We will thus not further discuss this result.

We also found an effect of age, with older dogs having a shorther latency than younger dogs (Pearson’s correlation, *r* = −0.35, *p* = 0.01, 95% CI = [−0.58–−0.06]).

## Discussion

The present study is the first to find evidence of behavioural synchronization of a dog toward its owner when both are moving freely in an enclosed room.

The first key finding was a strong location synchrony between dogs and their owners. When the owners started in the centre of the room and spent time there (i.e., in the control and still-move conditions), the dogs spent more time in the centre of the room. This is confirmed by the positive correlation between the time that dogs and owners spent in the centre of the room. Location synchrony was also evidenced by the fact that in the conditions where the owners spent most of their time on the sides of the room (i.e., in the still and move conditions), the dogs also spent more time on the sides of the room. This relationship was confirmed by positive correlations between the amounts of time that owners and dogs spent on each side of the room. Finally, the results showed that the dogs spent almost 80% of the time in close proximity to their owners. These effects did not depend on the dogs’ breed, sex, or age.

Second, the present study evidenced strong temporal and activity synchronies between the dogs and owners. Dogs switched to the same behaviour as their owners in 3.4 seconds on average. When their owner stayed still, dogs also stayed still. This was confirmed by a positive correlation between owners’ and dogs’ stationary time, as well as by the fact that the dogs’ stationary time in the control and still conditions was longer than in the still-move and move-still conditions, which in turn were longer than their stationary time in the move condition. Conversely, when their owner was moving, the dogs were moving too. Even more subtly, synchrony in gaze direction was also found. The more the owners looked toward the front of the room, the more the dogs did so as well (i.e., in the control and still conditions compared to other conditions, and cf. the positive correlation between gazing toward the front of room by the owners and the dogs).

The results thus confirmed all of our hypotheses. Like two people walking side by side^14^, when a person and a dog walk indoors, their movements are not independent, but synchronized.

Our results are in line with intraspecific findings. Dogs show behavioural synchronization with conspecifics in many activities, such as howling, sleeping, and moving^23^. Two other studies have observed synchronization when two dogs were running together: they influenced each other and the two synchronized their pace of running^24,25^. It has also been recently shown that dogs follow their conspecifics’ direction of walking during group departures^26^. The dogs were more synchronized (both location and activity synchrony) with their favourite social partners^26^. The authors proposed that it may reflect rules evolved for adaptation to the environment before domestication. As dogs evolved from wolves^27^, which usually follow their more experienced parents, dogs may be predisposed to follow their favourite partners and/or the more experienced individuals in their group^26^.

How might such a phenomenon appear at the interspecific level? Various mechanisms could be at play that would explain the non-conscious behavioural synchronization observed. One could argue that not talking to the dogs would have put them in an unatural setting, making them more stressed. We reject this possibility because dogs were behaving in a relaxed manner before we started the testing phases and throughout the experiment. Additionnaly, it was only during the 30 seconds of each condition that the owners were instructed not to talk or engage with their dogs, i.e. being ignored during only 30 seconds was not too stressful nor unusual for the dogs, as for example when the owners are answering calls while walking or not their dogs, dogs are indeed ignored. It is thus unlikely that dogs followed their owner because they were seeking proximity due to anxiety, as we controlled for stress-associated behaviour, and as all dogs were evaluated by their owners as behaving normally. Nevertheless, elevated physiological measures which could influence their behavior could have been at play. Even if not visibly stressed, dogs could have been more alert and could have been seeking proximity to owners as social support. We thus encourage further study to control for physiological parameters, or to test dogs in more familiar places, such as for example in their usual walking area.

However, another mechanism could explain the behavioural synchronization we found. It is known that dogs develop strong affiliative bonds with their owners^28–30^ and that often owners are the favourite social partners for their dogs^28,29^. Moreover, in daily life owners control access to the dogs’ food, leash, leisure time and various activities; the owners choose the timing, direction, and duration of walks, the place where the dog encounters other dogs, etc. Interestingly, it is known that in dogs, affiliation is of great influence in leadership^26^. The fact that the owner is mainly making decisions, such as initiating new directions of walks, may be considered as a type of leadership^31^. Additionally, leaders are often individuals possessing special skills about for instance the environment, which can be applied to humans over dogs in our societies^31^. Nevertheless one could argue that our results did not evidence an after-effect of affiliation, i.e. leadership, but instead local enhancement, a form of information transfer that can be observed in mixed-species stable groups^32,33^. In the broad sense, local enhancement is observed when the presence of a group mate at a specific location increases the probability that an observer goes to that location^34^. But in the stricto sensu, the display of this processes is linked to foraging contexts: local enhancement is how the presence of foragers at a location make it more obvious to others searchers^32,35^. In the present setting, we were very careful for the dogs not to be in a foraging context: the owners were not allowed to have food with them nor to provide food to the dog during the whole session (habitution, breaks, and testing conditions). In order to rule out the possibility that only local enhancement in the broad sense was at play, future studies might measure duration of ownership and owner’s attachment to their pet dogs to determine if dyads with stronger reported attachment would also show stronger synchronization. Another, probably more likely, explanation for location and activity synchrony between dogs and humans, is that dogs are reinforced for following their owners under many different circumstances. When dogs are on-leash, many owners tug on the leash whenever the dog trys to pull away, creating painful sensations that stop when the dog follows them: this is negative reinforcement for synchronizing their movements with those of their owners^36^. Whether dogs are on- or off-leash, many owners pet their dogs or give them treats for following them, or for coming back when called: this is positive reinforcement for synchronizing their movements with those of their owners. All of these phenomena may contribute to fostering the dog-human relationship and to making it beneficial for dogs to synchronize their movements (location, direction, walking speed) with those of their owner. Effect of learning through life experiences is confirmed by our findings that the older the dogs, the greater temporal synchrony we observed when switching activites. This latter hypothesis is also consistent with the gazing behaviour observed in the present study: dogs gazed where their owner gazed. This is congruent with recent studies on gaze following into distant space that emphazises the effect of training as well as daily experiences in gaze following behaviour^37,38^. This suggests that social cognition, learning and affiliation are involved in the synchronization of dogs’ behavior with that of the owner, confirming that the optomotor reflex hypothesis is less likely. Furthermore, as in humans, not moving in synchrony may be too costly for the dyad (e.g. decrease of cohesion and communication)^16^, or at least not being synchronized with their owners may be too costly for the dogs. Finally, it is worth mentioning that all dog-owner dyads tested were recruited over voluntary participation. It is thus possible that only owners with an interest in their dog’s behaviour, indicating a strong relationship with their dogs, participated in the study, explaining the high level of synchronization observed. That would be consistent with the finding that hormonal state synchronization has been found in dog-human dyads with a strong relationship^39^.

A question that finally arises is whether behavioural synchronization between dogs and humans is an invariant phenomenon across situations and different populations of owners and dogs. Given that in the present study dog-human non-conscious synchronization was found, even though dogs were observed in an unfamiliar enclosed room with a little time of familiarization with the environment, one could suggest that this phenomenon is likely to be robust and should be present in other contexts. For instance, it has been shown that sheep are more synchronized in larger spaces^40^. In open outside areas dogs spontaneously return towards their owners^31^ even if they are not very attentive to their owners^41^. However behavioural synchronization between humans and dogs was not the main focus of the two above-mentioned studies. It could thus be interesting to observe dogs in larger outdoor areas to see whether dogs’ movements still follow those of their owners as found in the present study. The results could possibly then be used in the development of strategies for managing dog behaviour. Additionnally, it would have been interesting to test the effect of the human’s sex. Due to unbalanced sex ratios, the design of our current study did not allowed us to properly test this parameter. However, since at least two studies have revealed that male and female owners do not behave in the same way with their dogs^42,43^, the effect of owner sex on the degree of behavioural synchronization would be justified to study. Furthermore, different populations of dogs with different affiliative bonds to humans, such as pet dogs and shelter dogs, are known to differ in their degree of sensitivity to humans’ behavioural cues (see ref.^44^ for a review). In humans, it is known that crawling/walking infants synchronize with their mothers, as proximity to the mother is critical for social development^45,46^. So, characterizing the effect of the degree of affiliation on dogs’ behavioural synchronization with humans is another issue of both theoretical and societal relevance. Investigating the attractive effect of the caregiver on shelter dogs, or of strangers on pet and/or shelter dogs, should shed light on the processes underlying behavioural synchronization.

The present study demonstrated for the first time the existence of dog-human behavioural synchronization when both partners move freely in an enclosed room. The phenomenon is so strong that it is visually observable (see movie S1): affiliated humans act as attractors for dogs. Pet dogs spontaneously synchronized their location and activity with their owners. This paper extends our understanding of the interspecific relationship between dogs and humans and adds data about the ability of dogs to read human communicative cues in general. We conclude that pet dogs act like their owners’ shadows.

## Methods

### Participants

Twenty-four molossoid and 24 shepherd pet dogs (12 males and 12 females in each group) were tested. Sample size was defined a priori on the basis of previous research (see ref.^47^). The dogs were between 1 and 11 years old (mean ± SE = 4.5 ± 0.41 years) and did not show any signs of health problems related to ageing (e.g., eye or joint problems) or behavioural problems (according to their owner’s reports). The testing room was novel to all dogs.

### Ethical note

The study was conducted in accordance to the legal requirements of France (where it was carried out), and the institutional guidelines of the Aix-Marseille Université, France. The owners all signed a consent form for study participation, and publication of identifying images. The dogs were not physically or psychologically harmed in the course of our study. All of the dogs were free to move in the room without physical constraints. The dogs did not undergo any physical intervention (such as blood or saliva sampling). After the test, all dogs returned home with their owners.

### Procedure

**Figure 4:**
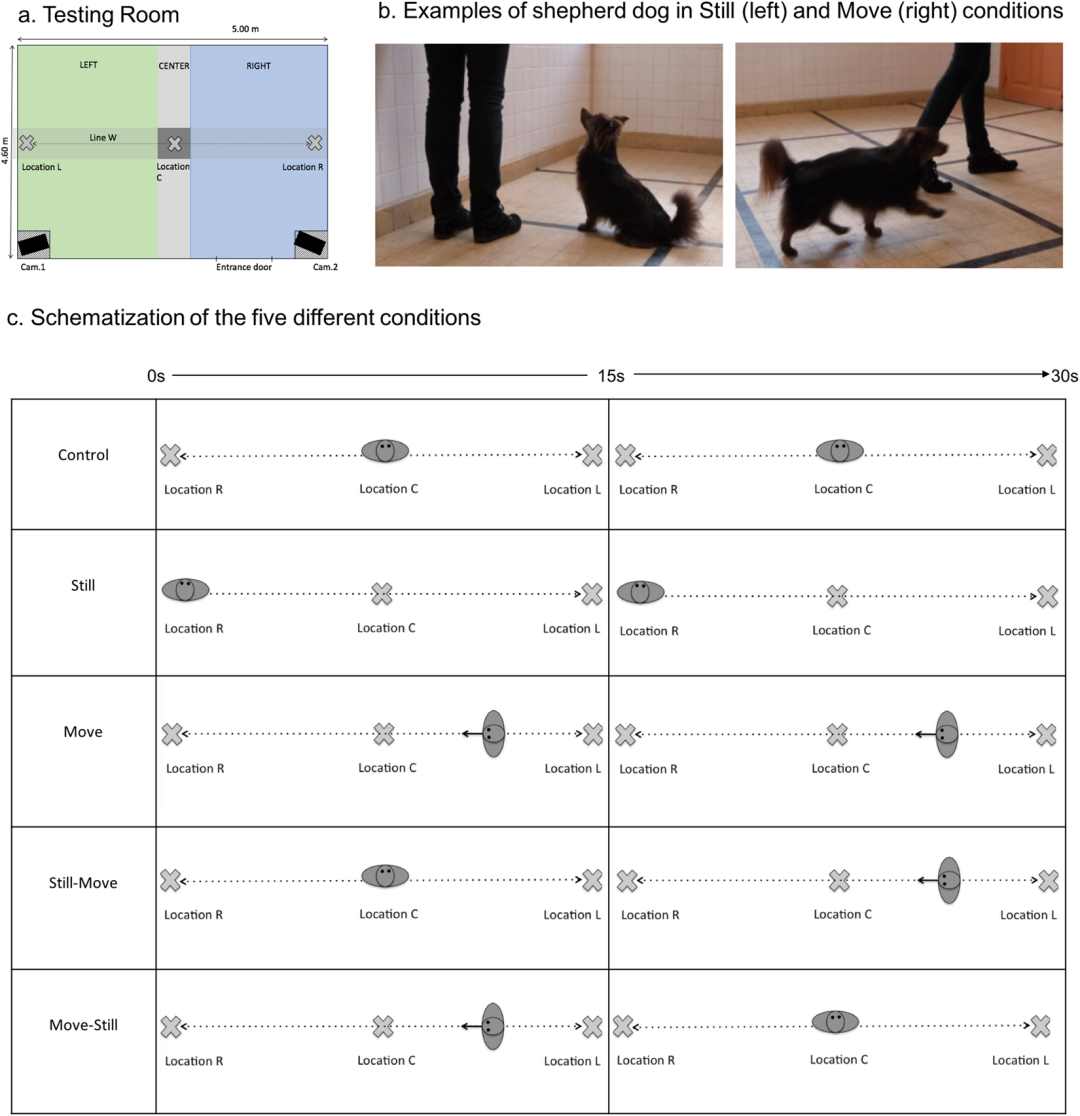
Experimental setting. Photography Credits: Charlotte Duranton. Cam = camera.

Dogs were tested in an unfamiliar empty quiet room (23 m^2^, Fig. 4a) in the National Veterinary School of Maisons-Alfort (France). At the beginning of the experiment, dogs were given 10 minutes to roam freely in the room in the presence of their owner and the experimenter. This allowed the dogs to become familiar with the space. Meanwhile, the experimenter explained the procedure of the test to the owners, with instructions on how to behave in each testing condition. All dogs were tested in all conditions, and the order of conditions was randomly assigned. The dog, its owner, and the experimenter then left the room. In all conditions, the owner then entered the room with the dog off leash, and walked to a predefined location. In the control condition, the owner went to location C and stayed still there for 30 seconds (see Fig. 4c). In the still condition, the owner went to location L or R – the side was randomly assigned but counterbalanced across dogs – and stayed still for 30 seconds (see Fig. 4b left, 4c). In the move condition, the owner went to location L or R – the side was randomly assigned but counterbalanced across dogs – and then started to walk along line W for 30 seconds; the back and forth walk ended wherever the owner was at the end of the 30-second period (see Fig. 4b right, 4c). In the still-move condition, the owner went to location C and stayed still for 15 seconds, then walked back and forth along line W for 15 seconds; the walk ended wherever the owner was at the end of the 15-second period (see Fig. 4c). In the move-still condition, the owner went to location C and walked back and forth along line W for 15 seconds, then stopped at location C and stayed still for 15 seconds (see Fig. 4c).

During the habituation phase, when the dogs were exploring the room, owners were authorized to speak to their pet and offer social support to the dogs when they felt it was necessary. Additionnally, during each break between the conditions, owners could talk and engage in any activity they wanted with their dogs to ensure that the dogs were comfortable. Owners had to behave as usual with their dogs.

Throughout the test, the dogs were off leash. When the owners were still, they were always looking towards the front of the room (see Fig. 4). During the testing conditions, owners were instructed not to show any emotional reaction, talk to their dogs, or look at them. Conditions were separated by a 10-minutes break. Owners could interact normally with their dogs during the habituation phase as well as each break between the testing conditions.

See Movie S1 to watch 10 seconds-long excerpts of each condition.

### Behavioural analysis

Two cameras recorded the movements of both dogs and owners. Main studied variables (6) were: time spent in close range (<1 m radius circle) with the owner, time spent stationary, time spent in the centre of the room, time spent moving, time spent gazing toward the front of the room, and latency before dogs’ switching to same activity as owners; cf. results below. Secondary variables, that were complement of the main variables or additional variables were: time spent moving, time spent on the right side of the room, time spent on the left side of the room, time spent on either side of the room, time spent gazing toward the left side of the room, time spent gazing toward the right side of the room, time spent gazing toward the sides of the room, time spent on line W, time spent gazing at the owner, latency before dogs’ switching to still in the move-still condition, latency before dogs’ switching to move in the still-move condition. Results for secondary variables are presented only in the Supplemental Material available. Table S1 in the Supplemental Material available presents a definition of each variable and details of the behavioural analyses are also provided in the Supplemental Material available.

### Statistical analysis

To analyze the potential effects of experimental condition, sex, age, and breed and any interactions between them on dogs’ behavioural responses, we used R (version 3.2.0). We used a linear mixed model for dependent data (LMER; normal distribution of the residuals was graphically checked) to test the effects of condition, breed, sex and age on all measures of dogs’ behaviour (details are provided in the Supplemental Material available). Where needed, we carried out post hoc comparisons with Holm-Bonferroni corrections for multiple tests. Where necessary, we used Pearson’s correlations to characterize the relationship between dog-related variables and owner-related variables. Effect size (Cohen’s *d* for LMER, and *r* coefficient for Pearson’s correlations) and 95% confidence intervals (CI) are provided.

### Data availability

The datasets generated during and/or analysed during the current study are available in the Open Science Framework repository, https://osf.io/hux6w/?view_only=1843f609097f4b929efa694cabe96646.

## Electronic supplementary material

Supplemental Material

Video S1
