# Supplementary material for: Interspecific behavioural synchronization: dogs exhibit locomotor synchrony with humans
Source: Sci Rep. 2017 Sep 28;7:12384. doi: 10.1038/s41598-017-12577-z (PMC5620060; doi:10.1038/s41598-017-12577-z)
Supplement: Supplementary file 1 — Supplemental Material [file 41598_2017_12577_MOESM1_ESM.doc]

**Interspecific behavioural synchronization: dogs exhibit locomotor synchrony with humans**

Charlotte Duranton, Thierry Bedossa & Florence Gaunet

***Supplemental Material***

**Supplemental Methods**

*Participants*

Dogs were recruited through veterinarian offices as well as online social media on the basis of volunteer participation from their owners (77% females). Subjects were all pet dogs that were familiar with the usual obedience tasks, such as sitting, laying down, and walking on a leash. The shepherd dogs were 8 Belgian shepherds, 2 Swiss shepherds, 2 German shepherds, 2 Shetland shepherd, 1 Rough Collie, 1 Border Collie, 1 Beauceron, 7 mixed shepherd breeds; and the molossoid dogs were 6 Cane Corso, 3 American bulldogs, 2 Great Danes, 2 Fila Braseiro, 2 Boxer, 1 Newfoundland, 1 Rottweiler, 1 Mastiff, 6 mixed molossoid breeds.

*Behavioural analysis*

All trials were recorded by two video cameras. The different locations were marked on the floor, and the room was marked out in 1m squares. Behaviours were then recorded with Actogram Kronos 2. The studied variables for all conditions are described in Table S1. To test the reliability of the behavioural coding, in addition to the coding of 100% of the behaviours by the first author C. D., a blind coder who was unaware of the hypotheses and aims of the study was trained to use Actogram Kronos, and then coded a randomly selected subset of 40% of the data. The resulting Pearson correlation coefficients were good (time spent in proximity to the owner: 98% agreement, *p* <  0.001; time spent on the right side of the room: 96% agreement, *p* <  0.001; time spent on the left side of the room: 99% agreement, *p* <  0.001; time spent at the centre of the room: 97% agreement, *p* < 0.001; time spent on line W: 96% agreement, *p* < 0.001; time spent still and moving: 99% agreement, *p* < 0.001 for both). Finally, visual inspection of all videos for stress associated behaviour was performed (body shake, self-grooming, yawn, licking lips, according to^19^ ), and owner were asked if they estimated their dogs stressed or not during the test.

Table S1: Description of studied variables.

| Category | Variable | Description of behaviour |
| --- | --- | --- |
| Location | Proximity | The dog was 1 metre or less away from the owner. |
|  | Dog Centre | The dog was in the centre of the room |
|  | Dog Right | The dog was on the right side of the room. |
|  | Dog Left | The dog was on the left side of the room. |
|  | Dog Sides | The dog was on the right or left side of the room (corresponds to average of variables Dog Right and Dog Left). |
|  | Dog Line W | The dog was on the central line of the room, where the owner walked when moving. |
|  | Owner Centre | The owner was in the centre of the room – Location C. |
|  | Owner Right | The owner was on the right side of the room – Location R. |
|  | Owner Left | The owner was on the left side of the room – Location L. |
|  |  |  |
| Activity  *Locomotor activity*  *Gazing activity* |  |  |
|  | Dog Move | The dog moved, i.e., walked, trotted, or ran in the room without interacting with its environment. |
|  | Dog Still | The dog was with all four paws still, whether lying down, sitting, or standing. |
|  | Owner Move | The owner was moving, i.e., walking along line W. |
|  | Owner Still | The owner was standing still. |
|  | Gaze Owner | The dog was gazing at (head + gaze oriented towards) the owner. |
|  | Dog Gaze Front | The dog was gazing toward (head + gaze oriented towards) the front of the room (see Fig. 1 for details). |
|  | Dog Gaze Right | The dog was gazing toward (head + gaze oriented toward) the right side of the room (see Fig. 1 for details). |
|  | Dog Gaze Left | The dog was gazing toward (head + gaze oriented toward) the left side of the room. |
|  | Dog Gaze Sides | The dog was gazing toward (head +gaze oriented towards) the right or left side of the room (corresponds to average of variables Dog Right and Dog Left). |
|  | Owner Gaze Front | The owner was gazing toward (head + gaze oriented toward) the front of the room. |
|  | Owner Gaze Right | The owner was gazing toward (head + gaze oriented towards) the right side of the room. |
|  | Owner Gaze Left | The owner was gazing at (head + gaze oriented towards) the left side of the room. |
| Temporality | Switch to Still | The dog switched to the same action as the owner, i.e. stayed still after the owner went still in the Move-Still Condition. |
|  | Switch to Move | The dog switched to the same action as the owner, i.e started to move after the owner started to move in the Still-Move Condition. |
|  | Switch to Action | The dogs switched to the same action as the owner in the Move-Still and Still-Move conditions (corresponds to average of variable Switch to Move and Switch to Still). |

*Statistical analysis*

Six variables were analyzed in a different way. Since the time spent by the dog on the right and the left sides of the room did not statistically differ (variables *Dog Right* and *Dog Left* respectively), we pooled them together to create a single variable for the time spent by the dog on the sides of the room (*Dog Sides*, see Table S1), which was analyzed using a linear mixed model for dependent data. The same analysis was performed on the time spent gazing toward the right or the left side of the room (the variables *Dog* *Gaze Right* and *Dog Gaze Left* respectively, see Table S1): they did not differ statistically, so we pooled them together to get the time spent gazing toward the sides (variable *Dog Gaze Sides,* see Table S1), which again was analyzed with a linear mixed model for dependent data. Since the latency before switching of action did not statistically differ (variables *Switch Still* and *Switch Move* respectively), we pooled them together to create a single variable for the time spent by the dog on the sides of the room (*Switch Action*, see Table S1) which was analyzed with a linear model for independent data.

**Supplemental Results**

Table S2: Descriptive data for all variables.

| Variable | Control | Still | Move | Still-Move | Move-Still |
| --- | --- | --- | --- | --- | --- |
| Proximity  Dog Centre  Dog Right  Dog Left  Dog Sides  Dog Line W  Owner Centre  Owner Right  Owner Left  Dog Move  Dog Still  Owner Move  Owner Still  Gaze Owner  Dog Gaze Front  Dog Gaze Right  Dog Gaze Left  Dog Gaze Sides  Owner Gaze Front  Owner Gaze Right  Owner Gaze Left | 25.31 ± 1.09  17.04 ± 1.85  6.93 ± 1.56  5.60 *±* 1.35  12.53 ± 1.84  10.45 ± 1.82  30.00 ± 0.00  0.00 ± 0.00  0.00 ± 0.00  6.80 *±* 1.04  23.18 ± 1.04  0.00 ± 0.00  30.00 ± 0.00  5.46 ± 0.80  8.61 ± 0.90  6.90 ± 1.00  6.34 ± 0.82  13.24 ± 0.98 30.00 ± 0.00  0.00 ± 0.00  0.00 ± 0.00 | 23.79 ± 1.29  1.94 ± 0.77  14.21 ± 1.98  13.84 *±* 2.02  28.05 ± 0.77  12.27 ± 1.71  0.00 ± 0.00  14.37 ± 2.18  15.62 ± 2.18  6.79 ± 0.99  23.20 ± 0.99  0.00 ± 0.00  30.00 ± 0.00  9.07 ± 1.07  8.00 ± 0.94  8.31 ± 0.97  6.20 ± 0.80  14.52 ± 0.92  30.00 ± 0.00  0.00 ± 0.00  0.00 ± 0.00 | 22.94 ± 0.83  4.47 ± 1.07  11.70 ± 0.77  13.79 *±* 0.89  25.49 ± 1.07  15.77 ± 1.16  0.00 ± 0.00  14.32 ± 0.23  15.65 ± 0.23  20.61 ± 1.06  9.38 ± 1.06  30.00 ± 0.00  0.00 ± 0.00  24.90 ± 0.63  1.10 ± 0.26  13.97 *±* 0.61  13.21 ± 0.57  27.18 ± 0.55  0.00 ± 0.00  14.72 ± 0.29  15.24 ± 0.29 | 22.73 ± 1.01  14.70 ± 1.31  7.52 ± 0.95  7.77 *±* 1.08  15.29 ± 1.31  12.39 ± 1.53  16.20 ± 0.06  6.82 ± 0.16  6.95 ± 0.18  12.92 ± 0.90  17.00 ± 0.92  15.00 ± 0.00  15.00 ± 0.00  16.63 ± 0.77  4.38 ± 0.66  9.98 ± 0.80  10.72 ± 0.73  20.71 ± 0.79  15.00 ± 0.00  7.08 ± 0.19  7.62 ± 0.21 | 24.41 ± .80  8.09 ± 1.21  9.39 ± 1.01  12.50 *±* 1.47  21.89 ± 1.12  16.46 ± 1.28  15.12 ± 0.06  7.33 ± 0.21  7.36 ± 0.26  14.82 ± 0.92  15.16 ± 0.92  15.00 ± 0.00  15.00 ± 0.00  15.65 ± 0.71  4.88 ± 0.73  10.42 ± 0.57  10.08 ± 0.64  20.50 ± 0.65  15.00 ± 0.00  7.40 ± 0.19  7.55 ± 0.18 |

Data presented in the table are: mean in seconds ± standard error. *N=*48 for all.

Table S3: Non-significant results for all variables.

| Dependent Variables | Independent variables | *F value* | Df | *P* |
| --- | --- | --- | --- | --- |
| Proximity | Condition  Sex | 5.83  0.02 | 4  1 | 0.21  0.88 |
|  | Breed | 1.92 | 1 | 0.16 |
|  | Age | 1.90 | 1 | 0.16 |
| Dog Centre | Sex | 0.97 | 1 | 0.32 |
|  | Breed | 0.95 | 1 | 0.32 |
|  | Age | 1.57 | 1 | 0.21 |
| Dog Right | Sex | 0.45 | 1 | 0.50 |
|  | Breed | 0.28 | 1 | 0.59 |
|  | Age | 0.77 | 1 | 0.38 |
| Dog Left | Sex | 0.03 | 1 | 0.85 |
|  | Breed | 0.23 | 1 | 0.63 |
|  | Age | 3.02 | 1 | 0.07 |
| Dog Sides | Sex | 0.82 | 1 | 0.36 |
|  | Breed | 1.22 | 1 | 0.26 |
|  | Age | 1.81 | 1 | 0.17 |
| Dog Line W | Sex | 0.38 | 1 | 0.53 |
|  | Breed | 0.10 | 1 | 0.75 |
|  | Age | 0.40 | 1 | 0.52 |
| Dog Move | Sex | 0.90 | 1 | 0.34 |
|  | Breed | 0.06 | 1 | 0.79 |
|  | Age | 0.04 | 1 | 0.83 |
|  |  |  |  |  |
| Dog Still | Sex | 0.95 | 1 | 0.33 |
|  | Breed | 0.06 | 1 | 0.80 |
|  | Age | 0.02 | 1 | 0.87 |
| Gaze Owner | Sex | 0.89 | 1 | 0.34 |
|  | Breed | < 0.01 | 1 | 0.98 |
|  | Age | 1.52 | 1 | 0.21 |
| Dog Gaze Front | Sex | 3.06 | 1 | 0.08 |
|  | Breed | 0.30 | 1 | 0.58 |
|  | Age | 1.05 | 1 | 0.30 |
| Dog Gaze Right | Sex | 0.19 | 1 | 0.65 |
|  | Breed | 1.14 | 1 | 0.28 |
|  | Age | 0.31 | 1 | 0.57 |
| Dog Gaze Left | Sex | < 0.01 | 1 | 0.99 |
|  | Breed | 0.10 | 1 | 0.74 |
|  | Age | 0.06 | 1 | 0.80 |
| Dog Gaze Sides | Sex | 0.20 | 1 | 0.64 |
|  | Breed | 0.58 | 1 | 0.44 |
| Switch to Action | Sex | 3.24 | 1 | 0.08 |

Results of the ANOVAs are provided.

We present below results of the secondary variables.

*Location synchronization*

*Time spent at the sides of the room*

We found that dogs spent significantly more time on the sides of the room in the still and move conditions than in the other conditions (see Table S4).

Consistent with these results, for all conditions pooled, dogs’ time spent on the right side of the room was significantly positively correlated with their owners’ time spent on the right side of the room (Pearson’s correlation, *r* = 0.59, *p* < 0.001, 95% CI = [0.50 – 0.66]). The same was true for the left side of the room (Pearson’s correlation, *r* = 0.62, *p* < 0.001, 95% CI = [0.53 – 0.69]).

*Time spent on line W*

We found that dogs spent more time on line W in the move and move-still conditions than in the control, still, and still-move conditions, but the effect disappeared after correction for multiple tests (see table S4).

Dogs’ time spent on line W was significantly positively correlated with owner’s time spent moving on line W, for all conditions pooled (Pearson’s correlation, *r* = 0.16, *p* = 0.010, 95% CI = [0.03 – 0.28]).

***Activity synchronization***

*Time spent moving*

Dogs spent more time moving in the move condition than in all other conditions (see Table S4). We also found a significant positive correlation between the time dogs spent moving and the time the owners spent moving for all conditions pooled (Pearson’s correlation, *r* = 0.60, *p* < 0.001, 95% CI = [0.52 – 0.68]).

*Time spent gazing towards the sides of the room*

As the time the dogs spent gazing to the right and to the left did not significantly differ (*t* test, *p* = 0.34), we pooled these two variables together (see Table S3). The results showed that dogs gazed significantly longer toward the sides in the move condition than in other conditions (see Table S4).

These results are confirmed by the finding of significant positive correlations between time spent by the owner gazing toward the right (or left) of the room and time spent by the dog gazing toward the right (or left) of the room respectively, for all conditions pooled (Pearson’s correlations: right, *r* = 0.39, *p* < 0.001, 95% CI = [0.28 – 0.49]; left, *r* = 0.48, *p* < 0.001, 95% CI = [0.38 – 0.57]).

*Time spent gazing at owner*

We found that dogs gazed at their owners for a significantly longer time in the move condition than in all other conditions (see Table S4).

*Stress-associated behaviours*

Dogs did not present stress-associated behaviours during the tests, except for one dog who performe allo-grooming once. All owners stated that their dogs were behaving normally.

Table S4. Significant and non significant results for the secondary variables.

| Dependant Variables | Results | Post-hoc comparisons | *χ^2^* | Df | *P* | Cohen’s *d* | 95% CI |
| --- | --- | --- | --- | --- | --- | --- | --- |
| Time at the side | Overall effect | **--** | 117.22 | 4 | <0.001 | -- | -- |
|  | Post-hoc | Control/Still | 68.52 | 1 | <0.001 | 1.04 | -19.33 – -11.71 |
|  |  | Control/Move | 37.82 | 1 | <0.001 | 0.57 | -17.50 – -8.42 |
|  |  | Control/SM | 1.87 | 1 | 0.17 | 0.15 | -6.86 – 1.34 |
|  |  | Control/MS | 19.28 | 1 | <0.001 | 0.47 | -13.74 – -4.98 |
|  |  | Still/Move | 3.80 | 1 | 0.051 | 0.17 | -5.42 – 0.31 |
|  |  | Still/SM | 77.21 | 1 | <0.001 | 0.93 | -15.71 – -9.80 |
|  |  | Still/MS | 23.56 | 1 | <0.001 | 0.52 | -8.74 – -3.58 |
|  |  | Move/SM | 45.77 | 1 | <0.001 | 0.76 | 7.14 – 13.27 |
|  |  | Move/MS | 6.06 | 1 | 0.014* | 0.26 | -6.58 – -0.63 |
|  |  | SM/MS | 18.14 | 1 | <0.001 | 0.47 | 3.45 – 9.75 |
| Time on line W | Overall effect | -- | 13.63 | 4 | <0.01 | -- | -- |
|  | Post-hoc | Control/Still | 0.84 | 1 | 0.35 | 0.11 | -5.85 – 2.21 |
|  |  | Control/Move | 6.18 | 1 | 0.012* | 0.21 | -9.88 – -0.75 |
|  |  | Control/SM | 0.97 | 1 | 0.032 | 0.14 | -5.92 – 2.05 |
|  |  | Control/MS | 7.44 | 1 | <0.01* | 0.22 | -11.00 – -1.02 |
|  |  | Still/Move | 3.33 | 1 | 0.067 | 0.20 | -0.39 – 7.39 |
|  |  | Still/SM | 0.00 | 1 | 0.94 | 0.01 | -3.13 – 3.36 |
|  |  | Still/MS | 3.93 | 1 | 0.047* | 0.19 | -0.18 – -8.56 |
|  |  | Move/SM | 4.03 | 1 | 0.044* | 0.23 | -0.03 – 6.81 |
|  |  | Move/MS | 0.17 | 1 | 0.67 | 0.04 | -2.64 – 4.02 |
|  |  | SM/MS | 5.78 | 1 | 0.016* | 0.27 | 0.63 – 7.52 |
| Time moving | Overall effect | -- | 216.04 | 4 | <0.001 | -- | -- |
|  | Post-hoc | Control/Still | 0.00 | 1 | 0.99 | 0.05 | -1.98 – 2.00 |
|  |  | Control/Move | 112.09 | 1 | <0.001 | 1.18 | -16.45 – -11.15 |
|  |  | Control/SM | 31.41 | 1 | <0.001 | 0.69 | -8.33 – -3.89 |
|  |  | Control/MS | 51.24 | 1 | <0.001 | 0.91 | -10.29 – -5.74 |
|  |  | Still/Move | 141.60 | 1 | <0.001 | 1.40 | 11.45 – 16.17 |
|  |  | Still/SM | 25.99 | 1 | <0.001 | 0.53 | 3.68 – 8.57 |
|  |  | Still/MS | 50.55 | 1 | <0.001 | 0.82 | 5.73 – 10.32 |
|  |  | Move/SM | 55.78 | 1 | <0.001 | 1.01 | 5.59 – 9.78 |
|  |  | Move/MS | 25.00 | 1 | <0.001 | 0.61 | -8.14 – -3.43 |
|  |  | SM/MS | 3.46 | 1 | 0.06 | 0.24 | -0.17 – 3.97 |
| Gaze at sides | Overall effect | -- | 204.34 | 4 | <0.001 | -- | -- |
|  | Post-hoc | Control/Still | 0.96 | 1 | 0.32 | 0.10 | -3.92 – 1.36 |
|  |  | Control/Move | 157.36 | 1 | <0.001 | 1.24 | -16.22 - -11.65 |
|  |  | Control/SM | 39.92 | 1 | <0.001 | 0.67 | -9.86 - -5.06 |
|  |  | Control/MS | 38.77 | 1 | <0.001 | 0.54 | -9.82 - -4.70 |
|  |  | Still/Move | 142.46 | 1 | <0.001 | 1.10 | 10.41 – 14.91 |
|  |  | Still/SM | 35.07 | 1 | <0.001 | 0.69 | -4.06 – 8.31 |
|  |  | Still/MS | 28.62 | 1 | <0.001 | 0.47 | -3.55 – 8.40 |
|  |  | Move/SM | 49.97 | 1 | <0.001 | 0.74 | 4.61 - 8.33 |
|  |  | Move/MS | 75.46 | 1 | <0.001 | 0.97 | -5.00 - -2.09 |
|  |  | SM/MS | 0.04 | 1 | 0.84 | 0.01 | -2.37 – 1.96 |
| Gaze at owner | Overall effect | -- | 444.78 | 4 | <0.001 | -- | -- |
|  | Post-hoc | Control/Still | 10.34 | 1 | <0.01 | 0.43 | -5.89 - -1.33 |
|  |  | Control/Move | 414.59 | 1 | <0.001 | 2.16 | -21.38 - -17.50 |
|  |  | Control/SM | 151.86 | 1 | <0.001 | 1.42 | -13.01 - -9.32 |
|  |  | Control/MS | 103.77 | 1 | <0.001 | 1.12 | -12.22 - -8.15 |
|  |  | Still/Move | 184.25 | 1 | <0.001 | 1.49 | 13.45 – 18.19 |
|  |  | Still/SM | 49.67 | 1 | <0.001 | 0.89 | 5.37 – 9.73 |
|  |  | Still/MS | 34.00 | 1 | <0.001 | 0.67 | 4.28 – 8.86 |
|  |  | Move/SM | 96.17 | 1 | <0.001 | 1.16 | 6.55 – 9.98 |
|  |  | Move/MS | 113.38 | 1 | <0.001 | 1.17 | -11.02 - -7.48 |
|  |  | SM/MS | 1.09 | 1 | 0.29 | 0.11 | -2.88 – 0.92 |

Results of the LMERs are provided. Post-hoc comparisons with an * were not significant after correction for multiple tests.

Time at the sides = time spent by the dogs at the sides of the room. Time moving = time spent by the dog moving. Gaze at sides = time spent by the dogs gazing at the sides of the room. Gaze at owner = time spent by the dogs gazing toward the owner. MS = Move-Still condition. SM = Still-Move condition.
